# Exploring proteomic signatures in sepsis and non-infectious systemic inflammatory response syndrome

**DOI:** 10.1371/journal.pone.0346812

**Published:** 2026-04-24

**Authors:** Adolfo Ruiz-Sanmartín, Vicent Ribas, David Suñol, Luis Chiscano-Camón, Laura Martín, Iván Bajaña, Juliana Bastida, Nieves Larrosa, Juan José González, María Dolores Carrasco, Núria Canela, Ricard Ferrer, Juan Carlos Ruiz-Rodríguez

**Affiliations:** 1 Departament de Medicina, Universitat Autònoma de Barcelona, Barcelona, Spain; 2 Intensive Care Department, Vall d’Hebron University Hospital, Vall d’Hebron Barcelona Hospital Campus, Barcelona, Spain; 3 Shock, Organ Dysfunction and Resuscitation (SODIR) Research Group, Vall d’Hebron Research Institute, Barcelona, Spain; 4 Eurecat, Centre Tecnològic de Catalunya, Digital Health Unit, Barcelona, Spain; 5 Department of Clinical Microbiology, Vall d’Hebron University Hospital, Vall d’Hebron Barcelona Hospital Campus, Barcelona, Spain; 6 Department of Genetics and Microbiology, Universitat Autònoma de Barcelona, Barcelona, Spain; 7 CIBERINFEC, ISCIII – CIBER de Enfermedades Infecciosas, Instituto de Salud Carlos III, Madrid, Spain; 8 Eurecat, Centre Tecnològic de Catalunya, Centre for Omic Sciences (COS), Joint Unit URV-EURECAT, Unique Scientific and Technical Infrastructures (ICTS), Reus, Spain; Pacific Northwest National Laboratory, UNITED STATES OF AMERICA

## Abstract

**Background:**

The search for new biomarkers that allow an early diagnosis in sepsis has become a necessity in medicine. This study aims to identify protein biomarkers that differentiate sepsis from non-infectious systemic inflammatory response syndrome (NISIRS), addressing the need for early sepsis diagnosis.

**Methods:**

Prospective observational study of a cohort of septic patients activated by the Sepsis Code and patients admitted with NISIRS, during the period 2016–2018. A mass spectrometry-based approach was used to analyze the plasma proteins in the enrolled subjects. Subsequently, using recursive feature elimination (RFE) classification and cross-validation with logistic regression, an association of these proteins in patients with sepsis compared to patients with NISIRS. The protein-protein interaction network was analyzed with String software.

**Results:**

275 patients were included (139 with sepsis and 136 with NISIRS. Plasma proteins were analyzed using mass spectrometry and evaluated through recursive feature elimination and cross-validation with a vector classifier. Twenty-five proteins showed statistically significant differences, with high diagnostic performance (sensitivity: 0.973, specificity: 0.920, accuracy: 0.960, AUC: 0.985). Fourteen proteins (VWF, PPBP, C5, C1RL, FCN3, SAA2, ORM1, ITIH3, GSN, C1QA, CA1, CFB, C3, LBP) were more associated with sepsis, while eleven (FN1, IGFALS, SERPINA4, APOE, APOH, C6, SERPINA3, AHSG, LUM, ITIH2, SAA1) were linked to NISIRS. The study found upregulation of several proteins in sepsis (C5, CFB, FCN3, PPBP, VWF, SAA2, ORM1, LBP) and downregulation of others (ITIH3, SERPINA4, AHSG).

**Conclusion:**

These findings highlight distinct proteomic patterns between sepsis and NISIRS. Advances in understanding these protein changes may allow for the identification of new biomarkers in the future.

## Introduction

Sepsis is known as a clinical syndrome where life-threatening organ dysfunction occurs due to a dysregulated host response to infection. The severity of sepsis varies significantly with the response and degree of organ dysfunction. Severe cases of sepsis, during which hypotension persists even after adequate fluid resuscitation and lactate levels > 2 mmol/L and the patient needs vasoactive support, are classified as septic shock [1]. Despite advances in diagnosis and treatment, sepsis remains one of the leading causes of morbidity and mortality worldwide, with a mortality rate ranging around 30–50% [2,3].

Current decisions regarding sepsis diagnosis and treatment are primarily based on Sequential Organ Failure Assessment (SOFA) and Quick SOFA (qSOFA), but their sensitivity and accuracy are known to be lacking [4,5]. C-reactive protein (CRP), procalcitonin (PCT), interleukin-6 (IL-6), and other biomarkers are also used for sepsis detection. Most of these biomarkers can reflect the immune system’s state and stages of the inflammatory cascade, being protein molecules with negatively regulated gene expression. CRP is frequently used to identify infections and sepsis. However, CRP cannot accurately reflect the severity of infection and sepsis because it increases during a minor infection or remains elevated even after the temporal course of the infection. Additionally, CRP levels can also rise during an inflammatory response to non-infectious events, trauma, tumorigenesis, or surgical interventions. These findings suggested that CRP lacks specificity as an early-stage sepsis biomarker [6,7]. PCT is likely the best-suited biomarker for infection at present, and it has even been proposed as a prognostic factor for sepsis progression [8] and a guide for antibiotic treatment duration [9]. However, it is hindered by false positives in non-infectious inflammation settings and a rather delayed induction (4–12 hours with a half-life of 22–35 hours) during the host’s response to infection [10,11]. Other biomarkers such as presepsin or pro-ADM have also been proposed as promising biomarkers in sepsis [12–13].

A deep understanding of the molecular and cellular mechanisms involved in sepsis is essential for more accurate and early diagnosis, as well as the development of new therapeutic strategies [14–15]. In this context, proteomics (a discipline of molecular biology that studies the complete set of proteins expressed in a cell, tissue or organ) has emerged

as a powerful and promising tool in the study of complex protein interactions underlying sepsis [16]. The use of high-throughput techniques such as liquid chromatography and mass spectrometry (MS) has enabled the identification of numerous candidate protein biomarkers for sepsis. However, their translation into clinical practice has been limited. The fundamental value of these proteomic studies lies in uncovering specific biomarker signatures and the pathogenic pathways involved, which constitutes a first step toward the future development of more precise diagnostic tools [17–19]. We hypothesized that there are proteomic patterns in patients with sepsis that differentiate them from patients with NISIRS. The objective of this study is to identify potential protein biomarkers of differential expression between sepsis and NISIRS.

## Materials and methods

### Study design and ethical approval

This is a prospective, observational, single-center study with two study populations conducted between April 1, 2016 and January 30, 2018. One study group with septic patients who met the criteria for activation of the Vall d’Hebron University Hospital in-hospital sepsis code [20] (ISC). This code could be activated in any hospital area (Emergency Department, wards, or ICU) upon clinical suspicion of sepsis. From these, only patients who subsequently met the SEPSIS-3 diagnostic criteria were included in the final sepsis cohort. The second study group included patients admitted to the Intensive Care Unit who met criteria for Systemic Inflammatory Response Syndrome (SIRS) without evidence of infection [21]. The study was approved by the Clinical Research Ethics Committee of Vall d’Hebron University Hospital [PR (AG) 11–2016, PR (AG) 336–2016, PR (AG) 210/2017], and written informed consent was obtained from all participants. The study fully adhered to the General Data Protection Regulation (Regulation (EU) 2016/679) and conducted in accordance with the ethical standards outlined in the 1964 Declaration of Helsinki and its subsequent amendments.

### Inclusion and exclusion criteria

The inclusion criteria for patients with NISIRS were adult patients ≥ 18 years who presented with two or more of the following variables: (1) white blood cell count >12,000/mm^3^ or <4,000/mm^3^, or >10% of immature neutrophils, (2) the presence of hyperthermia (axillary temperature >38.3ºC) or hypothermia (axillary temperature <36.0ºC), and/or tachycardia (>90 beats per minute), tachypnea (>20 breaths per minute or PaCO2 < 32 mmHg) and (3) absence of infection. The inclusion criteria for the septic patients group encompassed adult patients ≥ 18 years who met the criteria according to Sepsis-3 [1]: (1) presence of infection suspected or confirmed and (2) SOFA score ≥ 2 (Table 1).

**Table 1:** SOFA Score [22]. MAP: Mean arterial pressure, CNS: Central nervous system, GCS: Glasgow coma score. *Catecholamine doses = µg/Kg/min for, at least, 1 hour.

	0	1	2	3	4
Cardiovascular*	MAP ≥ 70 mmHg	MAP < 70 mmHg	Dopamine ≤ 5 or Dobutamine (any dose)	Dopamine 5.1–15 or Epinephrine <0.1 or Norepinephrine < 0.1	Dopamine >15 or Epinephrine ≥ 0.1 or Norepinephrine ≥ 0.1
**Respiratory**					
PaO2/FiO2 (mmHg)	> 400	301-400	201-300	101-200	≤ 100
**CNS**					
GCS Score	15	13-14	10-12	6-9	< 6
**Renal**					
Creatinine (mg/dL)	< 1.2	1.2-1.9	2.0-3.4	3.5-4.9	> 5
Urine output (mL/d)				< 500	< 200
**Liver**					
Bilirrubin (mg/dL)	< 1.2	1.2-1.9	2.0-5.9	6.0-11.9	≥ 12.0
**Coagulation**					
Platelets (x10^3^/mm^3^)	> 150	< 150	< 100	< 50	≤ 20

Exclusion criteria include non-adult patients, pregnant women or patients from whom a blood sample or written informed consent could not be obtained.

### Data collection and biomarker measurements

Following patient enrollment in the study, demographic data were recorded, and a venous or arterial blood sample was obtained for routine laboratory evaluation of laboratory analytical parameters. Blood samples were obtained at the time of sepsis or NISIRS diagnosis. Additionally, samples were collected for microbiological cultures in patients suspected of having sepsis. Clinical scores, such as SOFA, were retrospectively calculated whenever feasible at the time of enrollment. Measurements of CRP using an immunoturbidimetric test and lactate using an enzymatic color test were performed on these samples. The collected samples were frozen at −80ºC and stored in a Sepsis Bank of Vall d’Hebron University Hospital Biobank with appropriate ethics approval for subsequent analysis in accordance with clinical laboratory protocols. After patient recruitment was completed, the samples stored in the Biobank were delivered to the laboratory on February 7, 2018 for subsequent proteomic analysis.

### Proteomics analysis by mass spectrometry

The proteomic study was performed from plasma samples collected in Vacutainer K2E EDTA tubes (Becton Dickinson-Plymouth, United Kingdom) by the Proteomics and Metabolomics Area of the Center for Omic Sciences, a Joint Unit between Rovira I Virgili University and Eurecat (Reus, Spain).

#### Protein extraction and quantification.

Prior to proteomic analysis, depletion of the seven most abundant plasma proteins (albumin, IgG, antitrypsin, IgA, transferrin, haptoglobin, and fibrinogen) was performed to increase the number of identified/quantified proteins. Therefore, 12 μl of each sample was passed twice through the Agilent Technologies Human-7 Multiple Affinity Removal Spin cartridge and flow-through fractions were collected for proteomic analysis following the manufacturer’s protocol. Flow-through fractions were concentrated, and buffer was exchanged to approximately 100 µl of 6 M urea in 50 mM ammonium bicarbonate using 5K MWCO spin columns (Agilent 5185–5991).

#### Protein digestion and peptide 10-plex TMT labeling.

Thirty micrograms of total protein (quantified by Bradford’s method) were reduced with 4mM 1.4-Dithiothreitol for 1h at 37°C and alkylated with 8 mM iodoacetamide for 30 min at 25ºC in the dark. Afterwards, samples were overnight digested (pH 8.0, 37ºC) with sequencing-grade trypsin (Promega) at enzyme/protein ratio of 1/50. Digestion was quenched by acidification with 1% (v/v) formic acid and peptides were desalted on Oasis HLB SPE column (Waters) before TMT 10-plex labelling (Thermo Fisher) following manufacturer instructions.

To normalize all samples in the study along the different TMT-multiplexed batches used, a pool containing all the samples was labelled with a TMT-126 tag and included in each TMT batch. The different TMT 10-plex batches were desalted on Oasis HLB SPE columns before the nanoLC-MS analysis.

#### NanoLC-(Orbitrap)MS/MS analysis.

Labelled and multiplexed peptides were loaded on a trap nano-column (100 μm I.D.; 2 cm length; 5μm particle diameter, Thermo Fisher Scientific, San José, CA, USA) and separated onto a C-18 reversed phase nano-column (75μm I.D.; 15 cm length; 3μm particle diameter, Nikkyo Technos Co. LTD, Japan) on an EASY-II nanoLC from Thermo Fisher. The chromatographic separation was performed with a 180 min gradient using Milli-Q water (0.1% formic acid) and acetonitrile (0.1% formic acid) as mobile phase at a flow rate of 300 nL/min.

Mass spectrometry analyses were performed on an LTQ-Orbitrap Velos Pro from Thermo Fisher by an enhanced FT-resolution MS spectrum (R = 30,000 FWHM) followed by a data dependent FT-MS/MS acquisition (R = 15,000 FWHM, 40% HCD) from the most intense ten parent ions with a charge state rejection of one and dynamic exclusion of 0.5 min.

#### Protein identification/quantification.

Protein identification/quantification was performed on Proteome Discoverer software v.1.4.0.288 (Thermo Fisher). For protein identification, all MS and MS/MS spectra were analyzed using Mascot search engine (v.2.5). Mascot was set up to search SwissProt_2018_03. fasta database (557012 entries), restricting for Human taxonomy (20317 sequences) and assuming trypsin digestion. Two missed cleavages were allowed and an error of 0.02 Da for FT-MS/MS fragmentation mass and 10.0 ppm for a FT-MS parent ion mass were allowed. TMT-10plex was set as quantification modification and oxidation of methionine and acetylation of N-termini were set as dynamic modifications, whereas carbamidomethylation of cysteine was set as static modifications. The false discovery rate (FDR) and protein probabilities were calculated by Perclorator. For protein quantification, the ratios between each TMT-label against 126-TMT label were used and quantification results were normalized based on protein median. The results are a ratio of reporter ions abundance and are dimensionless.

### Statistical analysis

Demographic, clinical, and laboratory data were reported as mean ± standard deviation or median with interquartile range as appropriate, and categorical variables as numbers and percentages. The Student’s t-test was used for parametric quantitative variables, Mann-Whitney U test for non-parametric quantitative variables, and Chi-square test for qualitative variables. Statistical significance was determined at p < 0.05. The statistical analysis was performed using SPSS 18.0 software (SPSS Inc., Chicago, IL, USA).

In the proteomic study, prior to conducting any statistical analysis, each protein was standardized, and missing values were imputed using the k-nearest neighbor (KNN) method for proteins with less than 25% missing values. Proteins with major missing assignments were excluded from the study. The Kruskal-Wallis method with Benjamini-Hochberg false discovered rate (FDR) correction test (p < 0.05) was used to assess differences between distributions. Statistical analyses were conducted in Python 3.8 using the pandas, sklearn, scipy, and stats models libraries.

#### Protein selection.

Protein selection was carried out in five steps. In the first step data availability was assessed for completeness and consistency. In this step, data with a missingness percentage greater than 25% has been censored. This process filtered out 65 out of 177 proteins. After that, two different datasets have been generated: one where missing values have been imputed with the k-nearest neighbors (KNN) method and a second one with no imputation.

The second step consisted in a statistical analysis with the Kruskal-Wallis method with Benjamini-Hochberg false discovery rate (FDR) correction over the two datasets. The statistical analysis yielded the same list of 78 proteins with statistically significant expression values between sepsis and NISIRS.

The third step consists of a recursive feature elimination (RFE) with a logistic regression over the two datasets generated in the first step outlined above. RFE was applied with a 10-fold cross validation approach with stratified 80−20% splits for training and validation. The resulting predictions in validation between the two datasets were assessed with the MacNemar statistical test with a p-value of 0.3. Since there are no statistically significant differences between the results for the two datasets, it was decided to continue the experimental setup with the imputed dataset.

The fourth step consisted in assessing the discriminative power of the protein list obtained in the previous step. This step has been implemented with a logistic regression with a 10-fold cross validation approach with stratified 80−20% splits where accuracy, sensitivity, specificity, and AUC have been reported with 95% confidence intervals (95% CI). The logistic regression coefficients have also been reported with 95% confidence intervals, z-score, and p-values.

**Fig 1:**
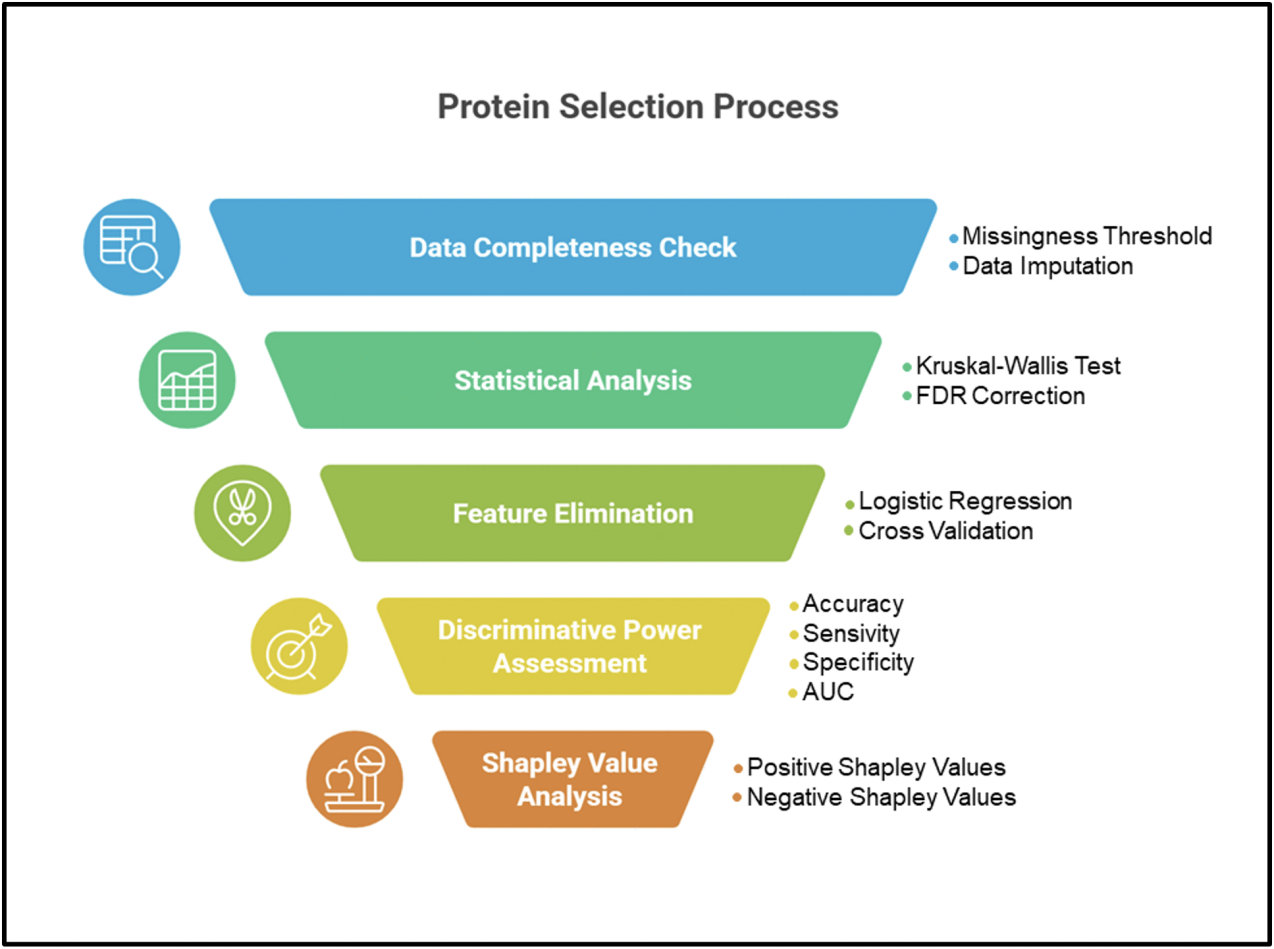
Diagram of protein selection.

In the fifth and final step, the coefficients of the logistic regression were analyzed using their additive Shapley explanations (SHAP values in summary). Proteins with positive Shapley values were associated with sepsis, while negative Shapley values were associated with NISIRS. The strength of association between the Shapley value and the outcome (sepsis and NISIRS) was measured by the magnitude of these Shapley values (Fig 1).

Protein selection was performed in Python 3.8 using the standard libraries pandas and scikit-learn. The protein-protein interaction network was analyzed using String v 11.0b software (https://string-db.org/).

## Results

### Characteristics of the study population

A total of 275 patients were included in this study; 139 patients in the sepsis group and 136 in the NISIRS group. The demographic and clinical data of the patients are shown in Table 2. For the sepsis group, the most common infection focus was urinary 48 (34.53%), followed by respiratory 47 (33.81%), and abdominal 44 (31.65%). In the NISIRS group, 107 (78.67%) patients had been admitted post-cardiac surgery, 13 (9.55%) were lung transplant recipients, 5 (3.67%) were liver transplant recipients, 4 (2.95%) had hemorrhagic shock, 3 (2.20%) were kidney transplant recipients, 2 (1.47%) were polytrauma patients, 1 (0.75%) had splenic hematoma, and 1 (0.75%) patient had acute pancreatitis.

**Table 2:** Characteristics of the study population. CRP: C-reactive protein.

Characteristics	Total (n = 275)	Sepsis (n = 139)	NISIRS (n = 136)	p
**Male** n (%)	162 (58.90)	83 (56.71)	77 (56.61)	0.53
**Age** years (m ± SD)	63.38 ± 15.61	63.9 ± 15.6	62.8 ± 15.5	0.54
**SOFA score** median (25^th^-75th)	5 (3-7)	7 (5-8)	3 (2-6)	<0.05
**Norepinephrine** n (%)	121 (43.68)	76 (54.67)	45 (33.08)	<0.05
**ICU admission** n (%)	206 (74.4)	70 (50.35)	136 (100)	<0.05
**Mechanical Ventilation** n (%)	177 (63.9)	41 (29.49)	136 (100)	<0.05
**Leucocytes x 10**^**6**^**/L** (mean ± SD)	14118 ± 9149	13501 ± 11021	14757 ± 661	0.25
**Platelets x 10**^**9**^**/L** median (25^th^-75th)	130.85 (116.0-227.5)	184.00(114.0-278.5)	157.00(119.5-195.2)	<0.05
**Lactate mmol/L** median (25^th^-75th)	1.9 (1.4-3.1)	2.5 (1.8-4.1)	1.5 (1.0-1.9)	<0.05
**CRP mg/dL** (mean ± SD)	14.61 ± 11.90	21.77 ± 12.58	7.58 ± 5.06	<0.05
**Hospital Mortality** n (%)	35 (12.6)	33 (23.74)	2 (1.4)	<0.05

### Proteomic study results

Initially, a total of 110 plasma proteins were identified and quantified by untargeted mass spectrometry for comparative proteomic analysis between NISIRS and sepsis. After quality control and missingness filtering, univariate statistical analysis identified 78 proteins as differentially abundant between both groups. From this set, a multistep feature selection procedure based on recursive feature elimination and logistic regression was applied to derive a reduced panel of 25 proteins with the highest and most stable discriminative power. This final protein panel achieved an accuracy of 0.960 (95% CI: 0.936–0.983), a specificity of 0.920 (95% CI: 0.859–0.980), a sensitivity of 0.973 (95% CI: 0.945–1.00), and an AUC of 0.985 (95% CI: 0.972–0.997). The analyzed proteins are presented in Table 3.

**Table 3:** Differentiated proteins analyzed between sepsis and NISIRS.

Proteins studied in sepsis and NISIRS patients	
**C1RL** - Complement C1r subcomponent-like protein **C3** - Complement C3c alpha’ chain fragment 1 **C5** - Complement C5 alpha’ chain **C6** - Complement component C6 **CFB** – Complement factor B Ba fragment **APOE** – Apolipoprotein E **APOH** – Beta-2-glycoprotein 1 **FCN3** - Ficolin-3 **GSN** – Gelsolin **SERPINA3** - Alpha-1-antichymotrypsin **SERPINA4** – Kallistatin **LBP** – Lipopolysaccharide-binding protein **ITIH2** – Inter-alpha-trypsin inhibitor heavy chain H2 **ITIH3** - Inter-alpha-trypsin inhibitor heavy chain H3	**PPBP** – Connective tissue-activating peptide III **VWF** – Von Willebrand antigen 2 **AHSG** - Alpha-2-HS-glycoprotein chain A **FN1** – Fibronectin **CA1** – Carbonic anhydrase 1 **LUM** – Lumican **SAA1** – Serum amyloid protein A **SAA2** – Serum amylod A-2 protein **ORM1** - Alpha-1-acid glycoprotein 1 **IGFALS** – Insulin-like growth factor-binding protein complex acid labile subunit **C1QA** – Complement C1q subcomponent subunit A

**Fig 2:**
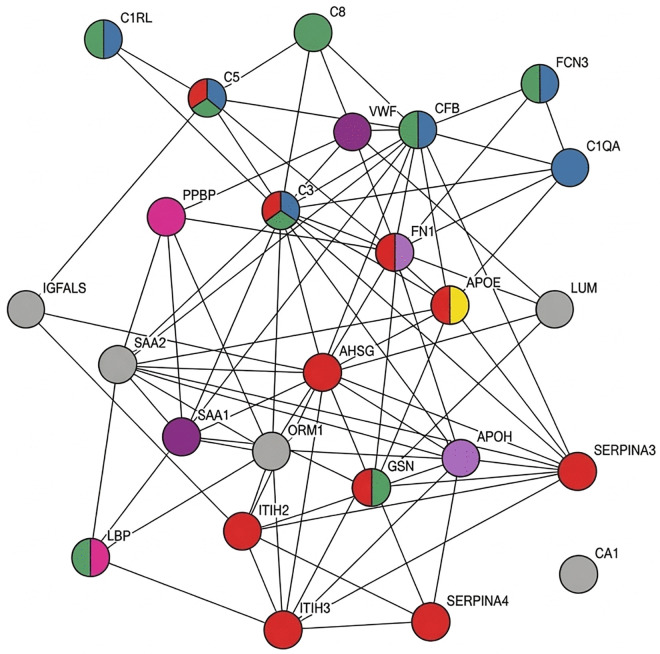
Strings network of physiological interactions between the different proteins analyzed (https://string-db.org/). Proteolysis (red), Innate immune response (green), Complement activation (blue), Response to lipopolysaccharides (pink), Blood coagulation (purple), Lipid metabolism (yellow), Other functions (grey).

Protein–protein interaction analysis using STRING showed that the proteins differentially expressed between sepsis and non-infectious SIRS do not appear in isolation but instead form a highly interconnected network. Within this network, several functional groups were identified, mainly related to the complement system and the acute-phase response, suggesting a shared functional organization among these proteins. Thus, of the 25 proteins found in this study, ten are involved in the regulation of proteolysis (SERPINA4, ITIH2, ITIH3, SERPINA3, FN1, APOE, C3, C5, GSN and AHSG), eight in innate immune response (LBP, FCN3, C3, C5, C6, GSN, CFB and C1RL), seven in complement activation (C1RL, C3, C5, C6, FCN3, C1QA and CFB), two in response to lipopolysaccharides (LBP and PPBP), four in blood coagulation (APOH, SAA1, FN1, VWF), two in lipid metabolism (APOE, APOH), and five proteins serve other functions (ORM1, IGFALS, CA1, SAA2 and LUM). The relationship among these proteins is presented in Fig 2.

**Fig 3:**
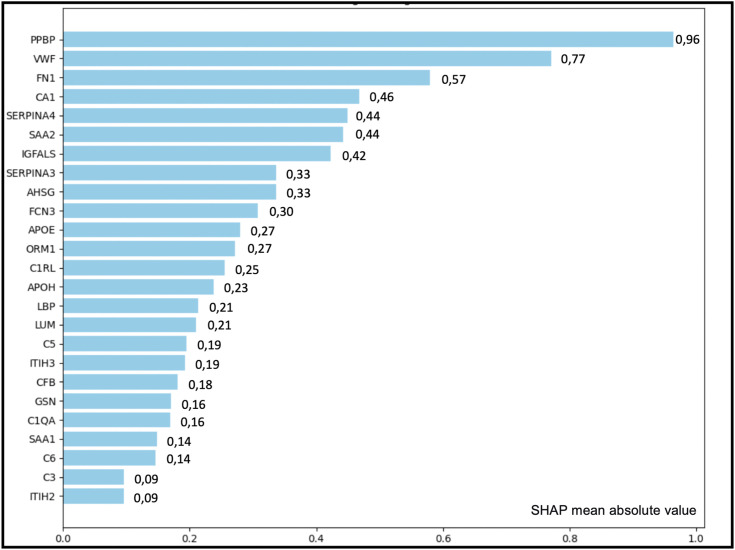
Mean absolute SHAP values of the logistic regression model. Mean absolute SHAP values indicate the relative contribution (association strength) of each protein to the model output when discriminating between sepsis and NISIRS. Higher values denote stronger influence on the classification.

When applying logistic regression model and analyzing its additive shap values, it was observed that the presence of 7 proteins with a higher association strength in the group of patients analyzed (sepsis and NISIRS patients): PPBP (0.96), VWF (0.77), FN1 (0.57), CA1 (0.46), SERPINA4 (0.44), SAA2 (0.44) and IGFALS (0.42). In contrast, proteins that show lower differential association strength between septic patients and patients with NSIRS are SAA1 (0.14), C6 (0.14), C3 (0.09) and ITIH2 (0.09) (Fig 3).

**Fig 4:**
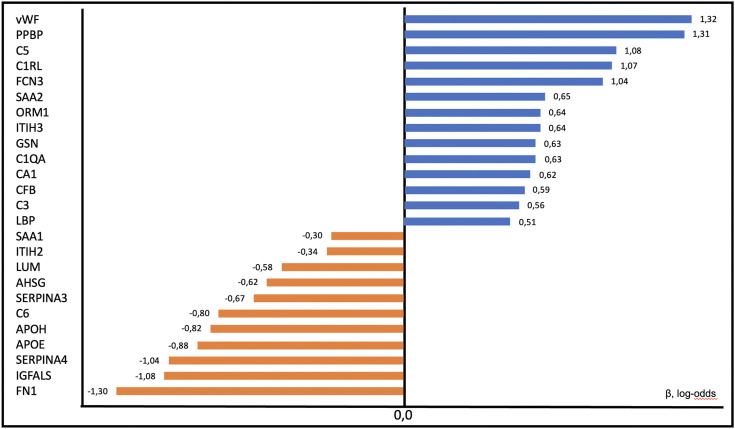
Logistic regression coefficients (β, log-odds scale) for the discrimination between sepsis and NISIRS. Positive coefficients indicate association with sepsis, while negative coefficients indicate association with NISIRS. Coefficients are reported on the log-odds scale.

After performing the analysis of logistic regression coefficients using their additive Shapley explanations, we detected that the main proteins with the greatest association with sepsis are VWF (+1.32), PPBP (+1.31), C5 (+1.08), C1RL (+1.07), SAA2 (0.65), ORM1 (+0.64) and ITIH3 (0.64). The proteins that presented the highest negative value and, therefore, present the greatest association with NISIRS are FN1 (−1.30), IGFALS (−1.08), SERPINA4 (−1.04), APOE (−0.88), APOH (−0.82) and C6 (−0.80) (Fig 4).

Finally, the comparative proteomic analysis performed using mass spectrometry coupled with the Tandem Mass Tag (TMT) relative quantification method revealed an upregulation of the proteins C5, CFB, FCN3, PPBP, VWF, SAA2, ORM1, LBP, and ITIH3. In contrast, the proteins SERPINA4 and AHSG exhibited downregulation.

## Discussion

This study identifies, through a proteomic approach, a distinct profile of 25 plasma proteins in patients that allows differentiation between sepsis and non-infectious Systemic Inflammatory Response Syndrome (NISIRS). The results highlight proteins such as von Willebrand factor (vWF), platelet basic protein (PPBP), complement component C5, and ficolin-3 (FCN3) as highly associated with sepsis. Conversely, proteins like fibronectin (FN1), insulin-like growth factor acid labile subunit (IGFALS), Kallistatin (SERPINA4), and apolipoprotein E (APOE) showed a stronger association with NISIRS. These protein signatures reflect not only a common systemic inflammatory response but also specific alterations in key pathways such as coagulation, complement activation, and innate immunity that appear more pronounced or qualitatively different in the presence of an infection. It is important to note that even though high-abundance protein depletion improves the detection of low-abundance species, it may result in the loss of carrier-bound proteins, which could explain the absence of some previously reported sepsis-associated proteins.

The primary contribution of this work lies in the identification of biomarkers with high discriminatory power between two clinical entities that share a similar phenotypic presentation but require radically different therapeutic management. Established biomarkers like C-reactive protein (CRP) or procalcitonin (PCT), while useful, lack optimal specificity in this context, as they can be elevated in severe inflammatory states of diverse etiology [6,7,10,11]. Our protein panel, with high diagnostic accuracy (AUC 0.985), suggests the existence of a more specific underlying molecular signature of the septic response. In particular, the strong association of vWF and PPBP with sepsis points to a distinctive endothelial and platelet activation, while the downregulation of serpins like SERPINA4 could indicate an exhaustion of anti-proteolytic mechanisms in sepsis, in contrast to NISIRS.

Translating these findings into the clinical setting could improve the diagnostic process in Emergency Departments and ICUs. Developing a rapid multiplexed assay based on this reduced set of proteins (e.g., vWF, PPBP, C5, FN1) would allow for rapid binary classification (septic vs. non-septic) with immediate implications for initiating or withholding empirical antibiotic therapy. In the longer term, quantifying these biomarkers could be integrated into clinical prediction scores (alongside SOFA/qSOFA) to create more robust decision algorithms, advancing towards a precision medicine model in critical patient management.

Our results are situated within a scientific context in which most proteomic studies in sepsis have used healthy controls as the control group [23–27]. This methodological choice, while valid for identifying disease-associated alterations, limits the specificity of the findings for distinguishing sepsis from other severe inflammatory states. This limitation is clearly illustrated when previously proposed biomarkers using that comparator are contrasted with our results obtained against a clinically more relevant group (patients with NISIRS). For example, APOE has been identified as a septic biomarker in studies comparing it to a healthy control population. Li M et al. observed an increase in APOE in sepsis with an AUC of 0.619 [28], and García-Obregón S et al. also reported it as altered [26]. Similarly, serum amyloid A1 (SAA1) is consistently elevated in septic patients versus healthy controls [23,25]. However, in our study, APOE showed a clear negative association (Shapley value −0.88), positioning it as a marker more characteristic of NISIRS, and SAA1 exhibited very low discriminatory power (Shapley value 0.14). This divergence reveals that the increase in APOE and SAA1 predominantly reflects the magnitude of the systemic inflammatory response and the metabolic alterations of critical stress – phenomena shared by sepsis and NISIRS – thus lacking the etiological specificity required for differential diagnosis in clinical practice. This interpretation could extend to other proteins like AHSG [27]. Collectively, these comparisons not only underline the expected divergences but also highlight how our approach, by using the appropriate comparator, is essential for advancing towards biomarkers with real clinical utility in distinguishing septic from non-septic inflammation.

Although multiple studies have observed distinct proteomic patterns in sepsis patients, few have compared them to groups with SIRS. The most significant one, conducted by Mi Y et al. [29], analyzed samples from 1,182 septic patients, 225 SIRS patients, and 152 healthy donors. Both studies share the use of high-resolution mass spectrometry, but their objectives differ. While Mi et al. characterize the broad proteomic landscape of sepsis and its relationship with severity subtypes, our study specifically focuses on the binary differentiation between sepsis and NISIRS. It is encouraging to observe that, despite these differences, there is significant overlap in proteins associated with sepsis versus non-infectious inflammation, including vWF, C1RL, C5, CFB, FCN3, LBP, SAA2, and ORM1. This convergence strongly supports the central role of these pathways (coagulation, complement, acute phase response) in the host’s specific response to infection.

In contrast, Shen et al. [30], in a study of sepsis and SIRS patients, reported no overlap with our top 25 proteins. This lack of concordance can be attributed to several factors. First, to technical and analytical differences: Shen et al. used immunodepletion and a three-dimensional mass spectrometry platform, while our method was based on a high-abundance depletion column and nanoLC-MS/MS analysis on an Orbitrap. Second, to a considerably smaller sample size (n = 25 per group versus our n = 139/n = 136), which reduces the statistical power and generalizability of their findings. And third, and perhaps most importantly, to possible uncharacterized heterogeneity in the definition and composition of their “NISIRS” group, which might have included conditions with very diverse inflammatory profiles, diluting specific proteomic signals.

These differences do not invalidate previous studies but rather contextualize their findings and highlight the importance of the research question. The strong overlap with Mi et al. [29] validates the core of our septic signature, while the differences with studies using healthy controls underline a key principle: then, the choice of comparator is decisive. Our work does not contradict that APOE or SAA1 are markers of severe inflammation; what it demonstrates is that they are not specific markers of infection when the differential diagnosis is posed against another equally critically ill patient with NISIRS. This distinction is fundamental for developing useful clinical tools. The strength of our approach lies precisely in using the clinically appropriate comparator, allowing us to filter out proteins elevated simply by “criticality” or “inflammation,” and to focus on those that capture the unique pathobiological signature of the response to a septic process, such as intense endothelial/prothrombotic activation (vWF), mobilization of specific myeloid mediators (PPBP), and particular regulation of the complement system (C5, C1RL) and its regulators (SERPINA4).

To assess whether transplant-related immunosuppression could confound the proteomic results, we performed a dedicated subgroup analysis comparing transplanted versus non-transplanted patients using a Mann–Whitney U test with Bonferroni correction. This analysis identified only three proteins (COL1A2, p = 0.026; APCS, p = 0.026; and COL1A1, p = 0.029) as differentially expressed between transplanted and non-transplanted individuals. Importantly, none of these proteins were part of the proteomic signature identified to discriminate NISIRS from sepsis.

When interpreting our proteomic differences, we must consider the potential confounding factor of disease severity. Our sepsis patients exhibited, on average, greater organ dysfunction (higher SOFA scores) than those with NISIRS. Recent studies have shown that many plasma proteins, including some of our findings, correlate with sepsis severity and are associated with subtypes of different prognoses [31,32]. Therefore, it is plausible that part of the signal we observe reflects varying degrees of systemic involvement.

Nevertheless, we believe the identified pattern also suggests etiological specificity. The strong association of sepsis with proteins involved in endothelial/prothrombotic activation (VWF, PPBP) and the complement cascade (C5, C1RL), in contrast to the association of NISIRS with proteins such as apolipoprotein E (APOE) or kallistatin (SERPINA4), points to qualitatively different humoral immune responses. This approach is consistent with the primary objective of our study, which is not to stratify severity within sepsis – a prognostic approach previously explored by our group [19] – but to provide a molecular signature for its differential diagnosis against sterile inflammation at the time of presentation.

This study has several limitations. First, this is a single-center study and our results may not be extrapolated to other patient populations. Second, in septic patients group, samples are collected upon sepsis code activation, and although they are taken early in the course of sepsis, we cannot rule out that all patients present the same stage of evolution at a pathophysiological level at the time of sample collection, which could affect the results. Third, our results only allow us to establish the degree of association of these proteins with sepsis in comparison to patients with NISIRS, without providing information on their specific concentrations in patients.

## Conclusion

The present study demonstrates that sepsis and non-infectious SIRS are characterized by distinct plasma proteomic profiles. By using a clinically relevant comparator group, our approach allows the identification of protein signatures that are more specifically associated with infection rather than with systemic inflammation or critical illness per se. The differential involvement of pathways related to endothelial and platelet activation, complement regulation, and innate immunity underscores fundamental pathophysiological differences between septic and non-septic inflammatory responses. These findings support the potential of proteomics-based biomarker panels to improve the differential diagnosis of sepsis in critically ill patients, although external and prospective validation will be required before clinical translation.
